# Nanomaterial Ecotoxicology in the Terrestrial and Aquatic Environment: A Systematic Review

**DOI:** 10.3390/toxics10070393

**Published:** 2022-07-14

**Authors:** Chiara Gambardella, Annalisa Pinsino

**Affiliations:** 1 Institute for the Study of Anthropic Impacts and Sustainability in the Marine Environment, National Research Council, 16149 Genova, Italy; 2 Institute of Translational Pharmacology, National Research Council, 90146 Palermo, Italy

**Keywords:** air, human, nanoparticles, soil, toxicity, water

## Abstract

This systematic review analyzes the studies available on the ecotoxicity of nanomaterials (NMs) in the environment to understand where future research should be addressed for achieving Agenda 2030 goals on sustainable development and environmental safety. We discuss the status of NMs ecotoxicological effects across different organisms that are representative of all natural environments (land, air, water). A total of 1562 publications were retrieved from the Web of Science (all databases) by using the search criteria “nanomaterials” and “ecotoxicology”; among them, 303 studies were included in the systematic review because they met any of the following criteria: (i) focalize on both search criteria; (ii) deal with terrestrial, or aquatic environment; (iii) address models (organisms, cells) for the nano environmental risk assessment and exposure. The knowledge gaps are identified together with novel insights that need to be further investigated to better understand the ecotoxicological environmental impacts of NMs.

## 1. Introduction

Nanomaterials (NMs) are defined as “natural, incidental, or manufactured material containing particles, in an unbound state or as an aggregate or as an agglomerate and where, for 50% or more of the particles in the number size distribution, one or more external dimensions is in the size range 1–100 nm” [1]. 

Due to their nanoscale size, NMs show unique chemical, biological and physical properties as compared with larger particles of the same material, which make them suitable for novel uses [2] in different fields, from medicine to food science, from drug delivery to cosmetics [3,4,5,6,7]. The application of NMs in different nano-based technologies has increased over the last few decades, thanks to the NMs properties, conveyed by their size, surface area, charge, and shape, that facilitate their use compared with their bulk or dissolved counterparts [8]. The wide application of NMs in our everyday life has inevitably resulted in their release and deposition into the environment, including in the soil, water, and air [9]. These different environmental compartments are strictly connected; thus, for example, NMs and all of the contaminants in the air do not remain confined in this matrix but can also be deposited in the soil and surface waters. Once in the different environmental matrices, NMs may interact with organisms; thus, the ecotoxicology of NMs in organisms belonging to different trophic levels has become an emerging and worldwide issue. In this regard, NMs have a great potential to affect human health, since they can reach humans directly or indirectly, through ingestion itself or along the food chain, adsorption, or inhalation [10]. Several studies have been published so far on NMs ecotoxicology in key species that characterize soil, water, and air, besides humans [11,12,13,14]. In the present review, we examined the current knowledge on the ecotoxicological impact of NMs in key species of all of the natural environments (e.g., soil, water, and air, including humans) and we identified the gaps concerning information on the impact of NMs along the trophic chain to be filled to predict the real bioavailability and biological risk of NMs in the next few decades (Agenda 2030). In this regard, NMs have a great potential to be employed in several daily applications, but a sustainable development towards the environment should be taken into consideration. Here we discuss the current knowledge on the ecotoxicological models used for predicting the toxicity of NMs, focusing on the literature retrieved from the Web of Science by the search criteria “nanomaterials” and “ecotoxicology”, and highlighting the information on: (1) the most studied species in this field; (2) the most NMs used; (3) the most analyzed environmental matrix; (4) the type of interconnection between NMs exposure and responses at different levels of organization (e.g., gene, cell, tissue, organism) and biomarkers; and (5) the limitation of the ecotoxicological information on NMs. To better understand the current research on the risk assessments associated with NMs and future trends, a network map was constructed through the systematic analysis of existing and selected articles. This map helped to discuss and highlight the future needs and suggestions in the NMs ecotoxicology field, considering the Agenda 2030 goals for sustainable development (e.g., ensure healthy lives and promote well-being for all at all ages; conserve and sustainably use the oceans, sea, and marine resources for sustainable development).

## 2. Materials and Methods

This review is based on a systematic search by using the Web of Science (WoS) citation database, an online portal of the following, the Institute for Scientific Information (ISI; currently Thomson Scientific, Philadelphia, PA, USA) citation databases: the Social Sciences Citation Index, the Arts, and Humanities Citation Index and the Science Citation Index [15]. It is an extensive and multidisciplinary database, covering many scientific journals, books, and proceedings in different fields, including natural sciences [16]. 

We queried “All databases” (including WoS Core Collection, BIOSIS Citation Index, Current Contents Connect, Data Citation Index, Derwent Innovation Index, KCI-Korean Journal Database, MEDLINE^®^, Russian Science Citation Index, SciELO Citation Index, Zoological Records), covering the period 1900–2021 (up to December the 31st), in the English language, by selecting different document types with the exclusion of “abstract”, “meeting” and “review articles”. As eligibility criteria, we retrieved the keywords ‘nanomaterial’ and ‘ecotoxicology’. Based on both title and abstract, the results were evaluated according to the following criteria:

Criteria 1-Does the search focus on both “nanomaterials” and “ecotoxicity”? (Yes/No);

Criteria 2-Does the result deal with terrestrial and aquatic environments? (Yes/No);

Criteria 3-Does the result address organisms as key targets? (Yes/No)

Only the results that fulfilled all of the criteria were included in the present review, analyzed, and discussed. Specifically, the documents were downloaded in Portable Document Format (PDF) for full-text assessment. The results were analyzed according to the publication year and the different environments. The results derived from the first search on the WoS database were refined by selecting only original articles (841 products), and only original articles that fulfilled the three criteria (303) were then used to create maps based on the network data of minimum occurrences of a term by using VOSviewer version 1.6.18 (Center for the Technology Studies of Leiden University). For this work, the counting method used to create the maps was set at the “full counting choose method” and at the threshold of “10 minimum number occurrences of a term” [17]. All of the terms were grouped into different clusters by circle size (bigger circles indicate higher importance). The closer the terms are located to each other, the stronger their relatedness [17,18]. The strongest links between terms are also represented by lines.

## 3. Results

**Figure 1:**
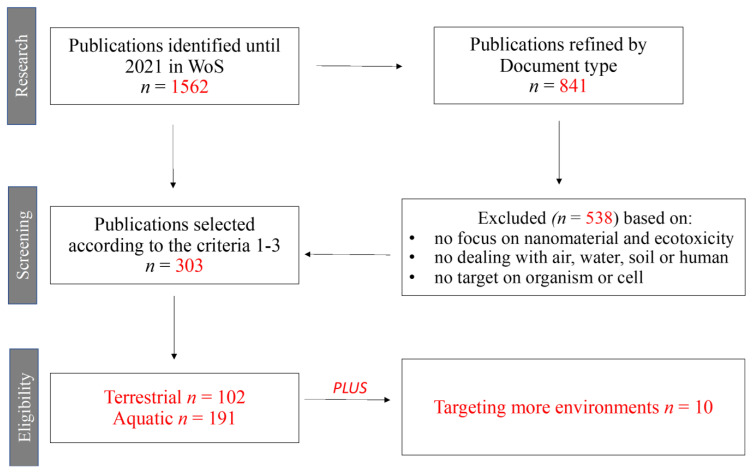
Flowchart of the selection process in this review.

Based on the title, abstract, and full-text availability, the WoS database released 1562 products reduced to 841, including only the original articles, of which a total of 303 papers, representing 19.4% of the total retrieved results, were found eligible for the present review according to the criteria one, two, and three (Figure 1).

**Figure 2:**
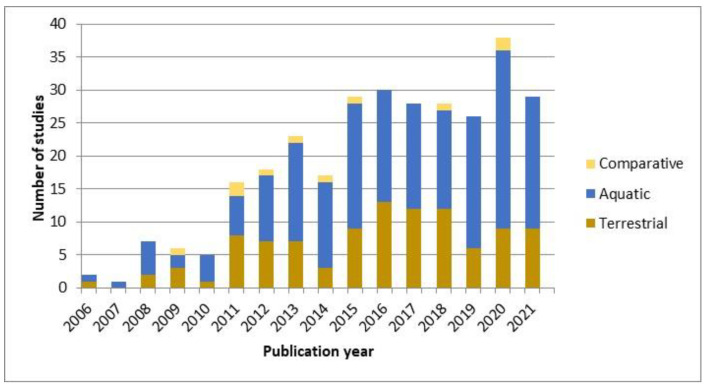
The number of studies published from 2006 to 2021 and selected for this review according to the eligibility criteria (*n* = 303), targeting aquatic, and terrestrial environments, and their combination (comparative studies).

An overview of the eligible publications (303) focusing on the impact of NMs on the terrestrial and aquatic environment from 2006 to 2021 is shown in Figure 2. Querying the WoS database using the search criteria “nanomaterials” and “ecotoxicology” and reporting only the eligible publications, we found that the interest in the topic has grown a lot in the last ten years, and the maximum number of studies was published in 2020 (*n* = 38). Moreover, the studies combining models from different environmental matrices (comparative studies) were addressed later, beginning in 2009 (Figure 2).

**Figure 3:**
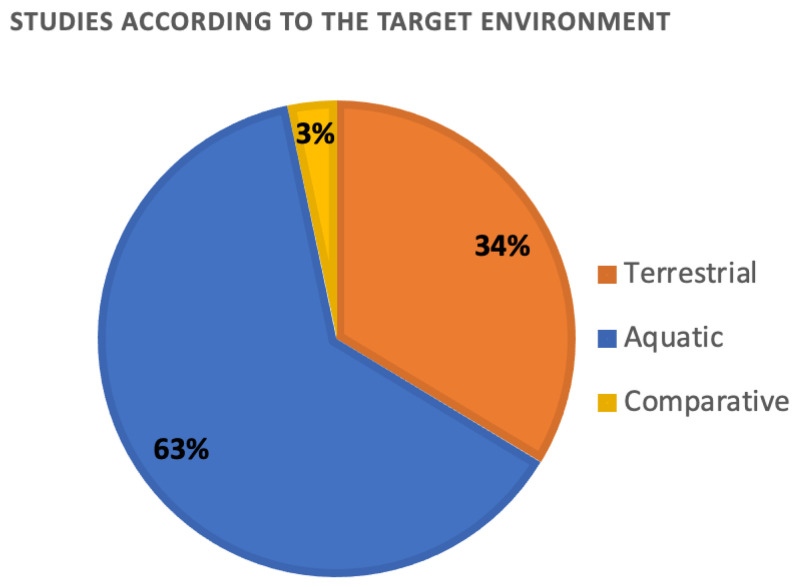
Percentage of selected studies categorized according to the terrestrial or aquatic environment. Comparative studies targeting both terrestrial and aquatic environments are also reported.

The percentage of the retrieved studies categorized for each environment is reported in Figure 3. The selected papers mainly focused on the ecotoxicological models for predicting the toxicity of NMs dispersed in aquatic environments (63%, *n* = 191). The studies on the terrestrial environment were fairly represented (34%, *n* = 102), whereas comparative studies were representative at very low percentages (3%, *n* = 10).

Besides reporting the main information on each of the 303 articles (e.g., article title, name of the journal where the article was published, year of publication, digital object identifier—DOI—number), the model organism and the NMs used were also added (Supplementary Tables S1–S3).

### 3.1. Web of Science Analysis

Based on the collected data, the selected studies on the aquatic and terrestrial environment were organized according to their compartment. Specifically, the aquatic environment was divided into three categories (freshwater, seawater, and comparative studies targeting both terrestrial and aquatic environments), and the ecotoxicological models were analyzed accordingly. The terrestrial environment was divided into four categories (air, soil, mammals, and comparative studies). A brief description, divided for each environment, is reported below.

- Aquatic environment

**Figure 4:**
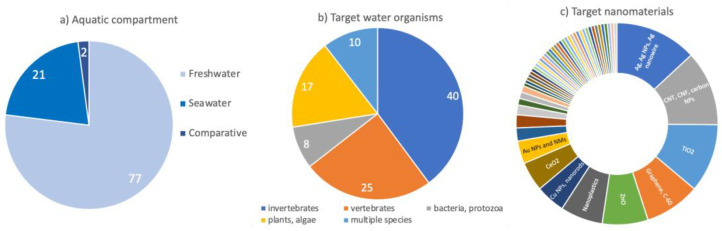
Overview of the selected papers targeting the ecotoxicity of nanomaterials in the aquatic environment classified according to the (**a**) different aquatic compartments; (**b**) the main target organisms; and (**c**) nanomaterials.

Water was the most representative environment in the selected papers (*n* = 191). The studies on the aquatic environment were mainly focused on freshwater (FW) species (77%, *n* = 141; Figure 4a), while those in the marine compartment only represented 21% (*n* = 40). The remaining 2% were comparative studies, addressing both FW and seawater (SW) species (*n* = 4).

Although all of the main trophic levels (decomposers, primary producers, primary and secondary consumers) were investigated, the retrieved studies focused mainly on invertebrates (40%), followed by vertebrates (25%), and plants or algae (17%) (Figure 4b; Table S1, Supplementary Materials). Among the invertebrates, a wide variety of organisms, including mollusks, crustaceans, cnidarians, and rotifers were used as ecotoxicological read-across models (for further details, see Supplementary Table S1). The studies on protozoans and bacteria and comparisons among two or more organisms were similarly represented with the lower percentage found (8% and 10%, respectively). The studies on aquatic organisms focused on a wide range of NMs, including mainly silver (Ag), titanium dioxide (TiO_2_) nanoparticles (NPs), carbon nanotubes (CNT), and fibers that exceeded 10% of the total. The carbon-based nanomaterials, graphene and fullerene, zinc (Zn), copper (Cu), and cerium (Ce)-based NPs were also well investigated, ranging from 4.7% to 8.89% (Figure 4c).

The ecotoxicity of NMs in invertebrates was mainly studied in crustaceans exposed to CNT, fullerene, graphene oxide, Ag, gold (Au), Cu, TiO_2_, ZnO, and CeO_2_ NPs, diamond, and polystyrene, in which the survival, reproduction, molting rate (e.g., in the marine brine shrimp and *Daphnia magna*), enzyme activities (e.g., cholinesterases, oxidative stress) and gene expression were investigated. Within the vertebrates, zebrafish, goldfish, and other species of commercial interest (e.g., tilapia, trout, carp, and sea bass) were used as models to assess the ecotoxicological impact of NMs. 

The acute and chronic toxicity of many NMs has been analyzed by using multi-biomarker and multi-endpoint approaches. It was found that the NMs, including metal, metal oxide, plastic NPs, CNTs, and graphene, can be responsible for altering viability, behavior (e.g., locomotor activity, feeding), morphology, development, physiology, hatching rate, and feeding in vertebrates at adult and larval stages. Changes in the activity of several enzymes (superoxide dismutase, catalase, malonaldehyde, glutathione-S-transferase, catalase, reactive oxygen species—ROS, DNA damage) were also investigated in several tissues (e.g., gill, liver, and intestine) of adult fish and in fish cell lines exposed to NMs [19,20].

NMs can also have toxic effects on primary consumers, by inhibiting the algal growth rate, reducing chlorophyll content, and altering the fatty acid composition, ROS, and lipid peroxidation (MDA) [21,22]. In addition, a direct action upon the algal membrane integrity was found in microalgae exposed to metal oxide NPs, such as TiO_2_ and silicon dioxide (SiO_2_) NPs [23]. 

Notably, the characteristics of the aquatic environment can influence the toxicity of certain NMs. For instance, TiO_2_ NPs are influenced by the light that enhances the toxicity of mussels and crustaceans [24,25]. Moreover, the major toxic impacts of CNT and nanoplastics (e.g., polystyrene) on mussels, algae, and bacteria were found to be influenced by salinity [26,27]. However, the salinity may facilitate NMs aggregation (mainly NPs), resulting in a lower level of toxicity. The NMs aggregates can interact with aquatic (both FW and SW) organisms of the same size of particles (e.g., algae), forming clusters [28,29]; the presence of large particle–algae agglomerates (e.g., TiO_2_, CNT) settle readily, dramatically depleting the concentration of available food for aquatic organisms. 

Overall, the ingestion, uptake, or bioaccumulation of nanomaterials in aquatic species was investigated in 11.5% (*n* = 22) of all of the studies based on aquatic models (Supplementary Table S1). It was found that exposure to or either ingestion or uptake of NMs can cause acute and chronic toxicity in FW and SW organisms, by inducing responses at different ecological levels, including organism (e.g., survival, reproduction, growth, behavior, feeding), cellular, and molecular ones (e.g., gene expression, enzyme activity, and biochemical responses).

- Terrestrial environment

**Figure 5:**
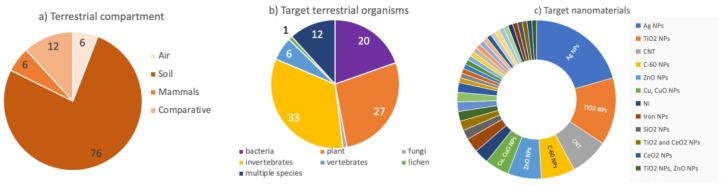
Overview of the selected papers targeting the ecotoxicity of nanomaterials in the terrestrial environment, classified according to (**a**) the different compartments; (**b**) the main target organisms; and (**c**) the selected nanomaterials.

A total of 102 studies targeting the toxicity of several NMs (e.g., polystyrene NPs, Ag, CeO_2_, iron oxide—Fe_3_O_4_, ZnO, CuO, Tin oxide—SnO_2_, SiO_2_, TiO_2_ NPs, CNT; see Supplementary Table S2) in the terrestrial environment fulfilled all of the criteria selected for this systemic analysis. Among the terrestrial compartments, most of the studies targeted soil model organisms (76%, *n* = 78; Figure 5a), followed by mammals and air-living organisms (6%, *n* = 6). The remaining 12% (*n* = 12) focused on comparative studies targeting different organisms (see comparative, Figure 5a) from the air and soil, including mammals.

**Figure 6:**
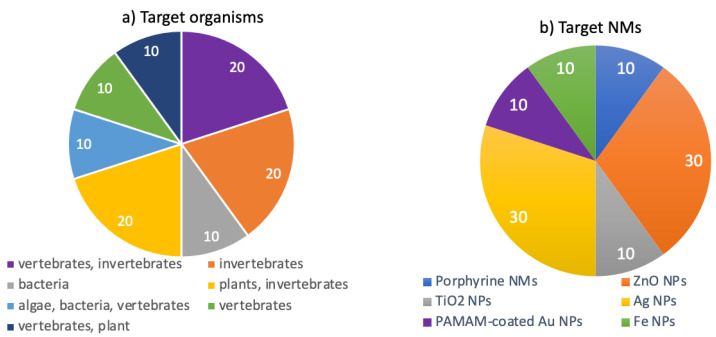
Overview of the selected papers targeting the ecotoxicity of nanomaterials in both aquatic and terrestrial environments, classified according to (**a**) the main target organisms and (**b**) nanomaterials.

Most of the studies focused on invertebrates (33%) and plants (27%), followed by bacteria (20%), vertebrates (6%), and lichens (1%, Figure 6b). About 12% of the remaining studies were comparative between two or more target organisms (e.g., plants–invertebrates, bacteria–protozoans, and fungi–yeasts). Most of the studies focused on Ag and TiO_2_ NPs ecotoxicity, representing 34.3% of the total articles (Figure 5c). The studies on carbon nanotubes, C-60, ZnO, and Cu NPs, were also represented, although to a lower percentage (<8%).

The ecotoxicity investigations of NMs towards soil organisms were the most represented. Among them, the terrestrial invertebrates (e.g., earthworms, oligochaetes) were deeply investigated, pointing to the effects on survival, weight change, and reproduction, including changes in the biochemical activity, such as the increase in MDA, and the reduction in glutathione content. The main NMs tested were the ZnO NPs, TiO_2_ NPs, and CNT. About 13.72% of the retrieved studies showed an NMs uptake (e.g., mainly cobalt—Co and Ag NPs) or an accumulation in soil organisms (mainly earthworms) that are easily excreted [30]. In plants, the main NMs tested were iron and TiO_2_ NPs, and the studies focused on the effects on growth, chlorophyll content seed germination, root elongation, shoot length, and leaf length. The studies on plants highlighted the capacity of certain NMs, such as CNT and fullerene, in the environment to enhance the bioavailability and translocation pattern of coexisting organic contaminants or currently used pesticides (e.g., carbamazepine, DDE, imidacloprid) in plants [31,32,33], or to absorb pesticides in edible plants, such as spinach and cauliflower [34]. 

Regarding bacteria, most of the retrieved studies analyzed the effects of NMs (e.g., TiO_2_, Ag, ZnO, Cu NPs) in terms of bacterial growth, enzyme activity, metabolism (e.g., denitrification, nitrification, respiration), and gene expression [35,36]. The NMs may act as vehicles to enhance or reduce the mobility of other elements of the soil or other contaminants, including pharmaceutical compounds, modifying their mobility in a natural soil–water environment. The NMs have little impact on soil microbiota, but their presence may increase the toxicity of the other contaminants altering the soil microbial population [37]. 

The ecotoxicity studies of NMs on the air compartment, fulfilling all of the criteria selected for this systemic analysis, focused mainly on CNT, fullerene, Ag, TiO_2_, zirconia—ZrO_2_, Fe-0, Fe_2_O_3_, manganese Oxide—Mn_2_O_3_, hafnium Oxide—HfO_2_, SiO_2_, aluminum oxide—Al_2_O_3_, CeO_2_ NPs, for a total of six studies. They targeted the ecotoxicity of NMs in the air in terms of atmospheric pollution and antimicrobial activities by using ecotoxicological models of bees, lichens, and bacteria (for further details, see Supplementary Table S2). Overall, NMs can be internalized in lichens by inducing physiological changes, they can reduce oxidative stress through the gene expression of antioxidative enzymes and changes in the redox state of the cells in bees, or enhance the photodynamic inactivation for Gram-negative bacteria under visible light irradiation [38]. 

The ecotoxicological effects of several NPs (e.g., Ag, Au, ZeO_2_, CeO_2_, TiO_2_, Al_2_O_3_, ZnO, graphene, and fullerene C-60) and CNTs were poorly investigated in mammals (only six studies), focusing on the cells, tissues, or whole organisms by using traditional and novel approaches. Among them, a mechanism-based structure–activity relationship (SAR) analysis to assess the human health hazard potential of NMs was reported [39].

- Comparative studies among models belonging to different environments

Ten studies targeting multiple compartments were found. They compared the ecotoxicity of several NMs in organisms belonging to terrestrial and aquatic environments, categorized according to the different targets (e.g., bacteria, invertebrates, and vertebrates; Figure 6a). Most of the studies focused on ZnO and Ag NPs, representing 60% of the total studies, while the remaining percentages focused on TiO_2_, iron NPs, porphyrin NMs, and polyamidoamine (PAMAM)-coated Au NPs (Figure 6b).

Differences in the sensitivity among the model species targeting the different environmental compartments were found after exposure to (i) PAMAM-coated AuNPs in mammalian cell lines, FW algae and marine bacteria [40]; (ii) ZnO NPs in plants, crustaceans, and insects [41,42,43]; (iii) Ag NPs in FW crustaceans, worms, and plants [44,45,46]. For example, while the Ag NPs are genotoxic for multiple species (e.g., mammals, plants, and aquatic organisms), magnetite NPs do not cause any potential risk to the environment, as indicated by a study that compares their effects in different models, such as human red blood cells, macrophage cell lines, onion root tips, marine crustaceans, and FW fish embryo (for further details, see Supplementary Table S3).

These studies recommend not only focusing on the inherent toxicity of NMs, but also considering their possible interactions with existing environmental contaminants.

### 3.2. VosViewer Analysis

**Figure 7:**
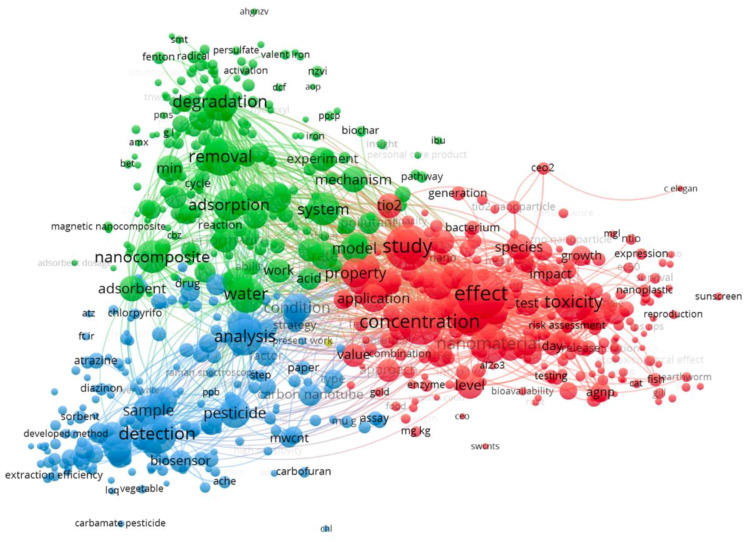
Map obtained by searching for keywords in 841 results using the VOSviewer software 1.6.18.

A total of 841 of the 1562 results were refined by typing the two keywords ‘nanomaterial’ and ‘ecotoxicology’ and excluding abstract, meeting, review articles, books, data papers, and editorial materials. The analysis of the main keywords found in the title and abstract of the 841 results was performed by VOSviewer software (1.6.18): 706 of 19,920 terms reached the threshold set at 10. The network visualization of the selected terms grouped by clusters and linked by lines is shown in Figure 7. 

The resulting 706 terms, which occurred at least 10 times in the title and the abstract fields, are distributed into three well-defined clusters, characterized by different colors, and focused on three main issues: effect, concentration, and study (red cluster); detection and analysis (blue cluster); degradation, removal, and adsorption (green cluster). 

**Figure 8:**
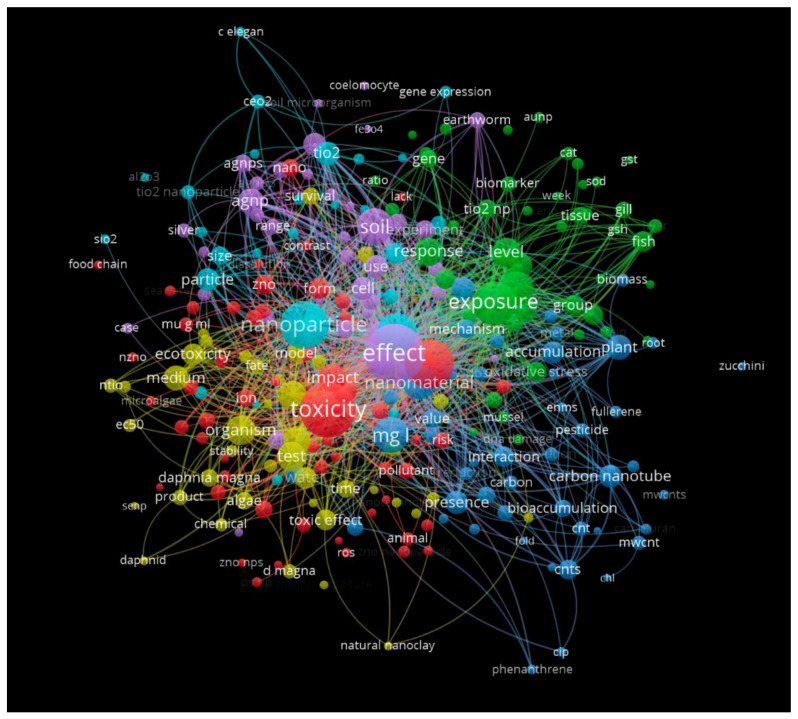
Map obtained by searching for keywords using the VOSviewer software 1.6.18 and selecting 303 articles.

Since the aim of this study was to evaluate the ecotoxicity of NMs focusing on ecotoxicological models for predicting the toxicity of NMs dispersed in all of the natural environments, the results were then analyzed according to the criteria described previously in the 303 selected papers. A total of 260 of the 8222 terms reached the threshold set at 10. The network visualization of the selected terms grouped by clusters and linked by lines is shown in Figure 8. 

The resulting 260 terms, which occurred at least 10 times in the title and the abstract fields, are distributed into five interconnected clusters characterized by different colors, from red to yellow. Each cluster focused on the three main issues: toxicity, impact, and nanomaterial (red cluster); exposure, level, and response (green cluster); nanoparticle, particle, and mechanism (light blue cluster); soil, effect, Ag NP (purple cluster); the smaller cluster, identified in yellow, focused on the organism, ecotoxicity, and test, where some target species (e.g., algae, aquatic invertebrates such as *Daphnia magna*) are reported. 

The map showing the toxicity of the NMs is related to the size, form, and concentrations (e.g., nano-size, ion, g/mL) of certain materials, including ZnO (red cluster). It impacts and poses a risk to microalgae and animals, affecting the food chain and inducing oxidative stress. The green cluster highlighted the interconnection among NMs exposure and responses at different levels of organization (e.g., gene, cell, tissue, organism), and biomarkers (e.g., GST, SOD, CAT). The light blue cluster highlighted the main MNs used (e.g., TiO_2_, Ag, CeO_2_ NPs), while the purple and the yellow revealed the environmental matrix and the species most studied in the field: soil and earthworms, water, and *Daphnia magna*, respectively.

## 4. Discussion

In this work, we presented an overview of the articles published so far on NMs ecotoxicology, based on the results extracted from WoS. Overall, we found that few ecotoxicological studies on NMs (<20% of the total results) fulfilled the selected criteria. A review of the Environmental Legislation for the Regulatory Control of NMs [47] reported similar results, highlighting the limitation of ecotoxicological information on NMs. In the last 15 years, a rapid growth in the number of studies on NMs ecotoxicity has started, with an increase since 2011, likely due to the definition of NMs from the European Commission [1]. While the studies published in the first few years targeted both terrestrial and aquatic environments, since 2012 the majority focused on the aquatic compartment. Although the Water Framework Directive (WFD) was established in 2000 [48], aimed at reducing and minimizing the concentrations of dangerous chemicals in EU waters, twelve years later, the European Commission revised this legislation, by incorporating NMs among the priority substances [49,50]. The inclusion of NMs under the WFD may explain the growing interest in NMs’ ecotoxicology in the water environment in the last decade, rather than in the terrestrial environment. In this regard, future research on the ecotoxicity of NMs in the terrestrial environment should be addressed in the next few years.

### 4.1. Significance of Invertebrates as Key Organisms to Assess NMs Ecotoxicity 

Most of the selected studies focus on the ecotoxicity of NMs in terrestrial and aquatic invertebrates. Invertebrates are more abundant than vertebrates [51]. Overall, invertebrates are being used extensively in laboratory tests to evaluate the toxicity of chemicals, contaminants, or environmental matrices (e.g., water, sediment). The use of invertebrates in ecotoxicological bioassays offers several advantages (e.g., easy maintenance and handling, short life span, possibility to be obtained by cryptobiotic or dormant eggs—cysts, high sensitivity to toxicants [52]) that may explain the extensive use of such organisms compared to vertebrates for the ecotoxicological assessment of NMs. 

The ecotoxicity of NMs in primary producers (plants, algae) and vertebrates were represented in the terrestrial and aquatic environments, although at a smaller percentage than that of invertebrates; conversely, the studies on NMs effects on humans/mammals are still scarce. To elucidate the impact of human exposure to NMs, advances in in vitro models (e.g., cell cultures) are a valid alternative [53], in line with the 3Rs (Replacement, Refinement, and Reduction of animals in research) principle. The uptake and translocation of certain NMs, such as NPs, have been successfully demonstrated in in vitro models [54]; despite that, only a few studies are available so far using this approach. The quality evaluation of human nanotoxicity problems has been addressed by the European Union FP-7 project GUIDEnano, which guided users to assess the nano risks of nanomaterial-enabled products throughout their life cycle, based on the few existing human toxicological and ecotoxicological studies [55]. Therefore, there is an urgent need to increase the number of studies aimed at investigating NMs ecotoxicity towards both humans and, in general, mammals. 

### 4.2. Nanoparticles Are the Most Studied NMs in the Environmental Compartments

New NMs are still being created for several applications [56]. To date, most of the retrieved studies focus on the ecotoxicology of NPs rather than the other NMs. For instance, Ag and TiO_2_ NPs were deeply investigated in terrestrial and aquatic organisms, while only recently has a growing interest started in the scientific community towards the ecotoxicological effects of CNT in biota [57]. Among the NPs, Ag NPs are emerging for use in next-generation applications in numerous fields, including nanomedicine, besides having been deeply investigated due to antibacterial properties and other favorable physical, chemical, and biological characteristics [58,59]. Moreover, even metal oxide NPs, such as TiO_2_ NPs, have been widely studied in both aquatic and terrestrial environments, due to their use in water treatments. When Ag and TiO_2_ NPs are suspended in aquatic solutions, they may transform from their pristine zerovalent state to surface-charge, ion-dissociated, and aggregated forms that may exert negative effects on the ecological systems, releasing toxic ions and ROS [60]. In the soil, TiO_2_ and Ag NPs are used in water treatment; they can be indirectly discharged into agricultural soils through irrigation or sewage–sludge applications, and directly as nano fertilizer [61,62], affecting the soil community.

Besides CNT, Ag, and TiO_2_ dioxide NPs, many other of the NMs (e.g., metal NMs, metal oxide NMs, fullerene, nanoplastics) have been extensively used in several applications, but they have not yet been investigated in the aquatic and terrestrial environment. Among these, the metal oxide NMs are in high demand for several reliable advanced technological applications (e.g., sensors, fuel cells, batteries, actuators, optical devices) due to their flexible mechanical, electronic, optical, electrical, catalytic, magnetic, and photochemical properties [63]. As an example, among the sensing NMs, CuO, SnO, ZnO, and NiO present high sensitivity, reproducibility, and stability, fast response/recovery time, and cheap and rapid fabrication processes. To fill this gap, we recommend that future studies should be addressed towards clarifying the ecotoxicity of the other NMs.

### 4.3. The Main Limitations of the Studies Come from the Past

Overall, proving the toxicity of NMs seems to have been the main concern among the retrieved studies. In this regard, most of the scientific articles test very high concentrations of NMs on organisms, targeting terrestrial and aquatic environments or both of them in order to find an ecotoxicological effect (e.g., 50% mortality—LC50; immobilization or growth inhibition—EC50 [21,41,64,65,66]). To date, most of the studies have used environmentally unrealistic concentrations to assess the NMs ecotoxicity. To meet the targets posed by the UN Sustainable Goal for the terrestrial and aquatic environments (e.g., SDG, Life on Land, Life below water, Good Health and Well-being), actions to prevent, monitor, and report on environmental pollution are urgently needed [67]. In this light, future studies should address more realistic NMs exposure scenarios (e.g., environmental concentrations measured in the terrestrial and aquatic environment) to clarify the ecotoxicological effects of NMs in biological models, and therefore either demonstrate NMs’ safety or predict the ecosystem impacts for a risk assessment [68]. Computational modeling, using a probabilistic approach, was suggested as a way of estimating the NMs environmental concentrations. However, these models inadequately consider the NMs’ fate in the environment. In addition, the absence of a selective analytical method in the complex matrixes makes difficult to determine if an increasing production of NMs leads to an increased NMs concentration in the environment.

### 4.4. NMs Behavior Can Be Transformed in the Environment

NMs can be transformed once in the environment, due to the interaction with natural parameters in the terrestrial and aquatic ecosystems (e.g., salinity, pH, temperature, organic matter) and to the presence of other contaminants (e.g., metals, chemicals, emerging compounds). The NMs can be a sink for organic and inorganic co-contaminants that may modify NMs’ physicochemical properties and behavior over time [60]; the interaction between NMs and other co-contaminants can impact NMs fate, distribution, and ecotoxicity, inducing additive, synergistic, or antagonistic responses [69,70]. On the other hand, NMs interact with a biological counterpart that forms a biological surface, coating the NMs. The bio-molecular coating or “bio-corona” is a highly dynamic structure and its composition changes over time, based on the absorption of organic matters (lipids, sugars, nucleic acids, metabolites, and particularly proteins) dispersed in the environment. The organic matters mask the NMs properties and develop a new biological identity that controls the interface with the environment, changing the behavior and fate of the NMs themselves. Consequently, the NMs in the environment undergo a transformation process (aging), including chemical transformation, aggregation, agglomeration, disaggregation, and deagglomeration. The interplay between all of these processes will determine the ecotoxicological potential of the NMs [8]. 

In this regard, most of the studies report on the ecotoxicological effects of organisms due to single NMs, rather than focusing on the co-exposure of NMs and other compounds, that may increase or decrease the potential toxicity of NMs towards aquatic or terrestrial organisms. We, therefore, encourage studies characterizing the use of multiple stressors to better understand the NMs behavior—in terms of toxicity—in organisms belonging to aquatic and terrestrial organisms. 

### 4.5. Need to Use a Multidisciplinary Approach to Evaluate NMs Ecotoxicity as a Whole 

Multi-stressor studies typically involve a set of organisms to baseline levels of all of the stressors based on environmental concentrations or simulating future conditions. They can be performed on single or multiple species, although the latter are potentially more complete, realistic, and sensitive than single-species-based ecotoxicological tests. Most of the retrieved studies of this review focus on bioassays on single rather than multiple species (namely comparative) to detect NMs ecotoxicity. The ecotoxicological batteries include the use of several species belonging to different taxonomic and trophic levels in the exposure experiments, allowing for quantifying acute biological effects [71]. The European legislative framework supports a multidisciplinary strategy of environmental monitoring, because the environment is a complex system of abiotic and biotic interactions [72]. Thanks to a multidisciplinary weight of evidence (WOE) approach, it is possible to integrate information from different Lines of Evidence (LOEs), assigning a different weight to each LOE, thus improving the ability to interpret alterations of environmental conditions and assess the probability of hazards [73]. By considering chemical analyses and ecotoxicological bioassays as different LOEs through a quantitative integration, it will be possible to convert complex scientific information into simple hazard indexes, easily understandable by policymakers and environmental managers, that can facilitate and orientate the more appropriate decisions on environmental NMs management. In our era, it is necessary to point to a rational science-based approach to minimize the risk for health, focusing on developing strategies for sustainable (safe) nano-production, promoting health policies, and embracing actions at an individual and social level. Since NMs should be as safe as possible for the environment and human health, the nanotechnology world must focus on a “safer-by-design” approach, based on three major challenges: (1) identify and develop less hazardous NMs (safer products by design); (2) evaluate the risks during consumer usage (safer use of products); (3) minimize waste (safer industrial production discharge) [74]. Thus, this approach focuses on designing NMs, promoting knowledge and learning, and minimizing risks at all of the stages, from manufacture to usage to the release on the environment.

## 5. Conclusions

This review identified the knowledge gaps that need to be further investigated in light of the Agenda 2030 goals. We found out that: (i) NPs are the most studied NMs in the environmental compartments, probably since these NMs satisfy several industrial and domestic purposes; (ii) aquatic organisms are the most investigated, probably because the aquatic environments are the most basic ways to deal with the contaminants; (iii) invertebrates are the most ecotoxicological models used for predicting the toxicity of NMs, probably because are easy to manipulate and do not present ethical issues; (iv) the whole organism is the major focus, probably because the approach is easy and fast. 

What should we do? Future research needs to: 

(1) address the ecotoxicity of NMs in the terrestrial environment, as well as on mammals; 

(2) clarify the ecotoxicity of metal and metal oxide NMs, fullerenes, nanoplastics, and other carbon-based NMs;

(3) test environmentally realistic concentrations;

(4) use a multidisciplinary approach to evaluate NMs ecotoxicity as a whole, by taking into account the multifactorial and changing behavior of NMs.

## Data Availability

Data supporting reported results can be found within the supplementary documents.

## Supplementary Materials

The following supporting information can be downloaded at: https://www.mdpi.com/article/10.3390/toxics10070393/s1, Table S1: The main information on the retrieved studies for the aquatic environment, including freshwater or marine compartment, target organisms, endpoint/responses, nanomaterial, uptake/accumulation, article title, Journal, Published year, DOI. Table S2: The main information on the retrieved studies for the terrestrial environment, including target organisms, endpoint/responses, nanomaterial, uptake/accumulation, article title, Journal, Published year, DOI. Table S3: The main information on the retrieved studies for the comparative studies targeting both terrestrial and aquatic environments, including target organisms, endpoint/responses nanomaterial, uptake/accumulation, article title, Journal, Published year, DOI.
